# Challenges in LncRNA Biology: Views and Opinions

**DOI:** 10.3390/ncrna10040043

**Published:** 2024-08-01

**Authors:** Donald A. Adjeroh, Xiaobo Zhou, Alexandre Rossi Paschoal, Nadya Dimitrova, Ekaterina G. Derevyanchuk, Tatiana P. Shkurat, Jeffrey A. Loeb, Ivan Martinez, Leonard Lipovich

**Affiliations:** 1 Lane Department of Computer Science and Electrical Engineering, West Virginia University (WVU), Morgantown, WV 26506, USA; 2 Department of Bioinformatics and Systems Medicine, University of Texas Health Science Center, Houston, TX 77030, USA; xiaobo.zhou@uth.tmc.edu; 3 Department of Computer Science, Bioinformatics and Pattern Recognition Group, Federal University of Technology—Paraná—UTFPR, Curitiba 86300-000, Brazil; paschoal@utfpr.edu.br; 4 Rosalind Franklin Institute, Harwell Science and Innovation Campus, Didcot OX11 0FA, UK; 5 Department of Molecular, Cellular and Developmental Biology, Yale University, New Haven, CT 06520, USA; nadya.dimitrova@yale.edu; 6 Department of Genetics, Southern Federal University, Rostov-on-Don 344090, Russia; derevyanchuk@sfedu.ru (E.G.D.); tshkurat@sfedu.ru (T.P.S.); 7 Department of Neurology and Rehabilitation, The Center for Clinical and Translational Science, The University of Illinois NeuroRepository, University of Illinois, Chicago, IL 60607, USA; jaloeb@uic.edu; 8 Department of Microbiology, Immunology & Cell Biology, WVU Cancer Institute, West Virginia University (WVU) School of Medicine, Morgantown, WV 26505, USA; ivmartinez@hsc.wvu.edu; 9 Shenzhen Huayuan Biological Science Research Institute, Shenzhen Huayuan Biotechnology Co., Ltd., Shenzhen 518000, China; 10 Center for Molecular Medicine and Genetics, School of Medicine, Wayne State University, Detroit, MI 48201, USA; 11 College of Science, Mathematics and Technology, Wenzhou-Kean University, Wenzhou 325060, China

**Keywords:** lncRNAs, cancer, COVID-19, lncRNA datasets, deep learning, large language models

## Abstract

This is a mini-review capturing the views and opinions of selected participants at the 2021 IEEE BIBM 3rd Annual LncRNA Workshop, held in Dubai, UAE. The views and opinions are expressed on five broad themes related to problems in lncRNA, namely, challenges in the computational analysis of lncRNAs, lncRNAs and cancer, lncRNAs in sports, lncRNAs and COVID-19, and lncRNAs in human brain activity.

## 1. Introduction

This mini-review collates selected views and opinions on certain topics in the area of long non-coding RNA (lncRNA) biology, bioinformatics, genomics, and therapeutics, from the perspectives of several keynote, plenary, and invited speakers, panelists, and organizers of the IEEE BIBM 3rd Annual LncRNA Workshop, which was held in Dubai, UAE, in December 2021, with both remote and in-person participation. As a mini-review, this article is not intended to be either comprehensive or complete, nor does it cover all the important issues surrounding today’s lncRNA research. Contributors were asked to write briefly on a topic of their choice as it related to their presentations or the discussions at the Workshop. This mini-review touches on five broad themes chosen for their relevance and appeal to the lncRNA community: challenges in the computational analysis of lncRNAs, lncRNAs and cancer, lncRNAs in sports, lncRNAs and COVID-19, and lncRNAs in human brain activity.

Given the focus on discussion and presentations at the 2021 Workshop, we acknowledge the limited scope of this mini-review, as it could not cover various equally important topics related to lncRNA—for instance, the accurate prediction of lncRNA targets and functions; tissue, cell type, and context specificity of lncRNAs and their roles in development, differentiation, and tissue homeostasis; roles of lncRNAs in cell communication and immune response; lncRNA function in the brain beyond epilepsy; the relationship of the lncRNAome to the conundrum of bifunctional RNAs; and other themes.

The views and opinions expressed are those of the authors and do not necessarily represent those of the IEEE or BIBM. Not all invited speakers contributed to this mini-review.

## 2. Current Challenges in the Computational Analysis of LncRNAs

*Alexandre Rossi Paschoal, Federal University of Technology—Paraná—UTFPR, Brazil; Rosalind Franklin Institute, Harwell Science and Innovation Campus, Didcot, UK,*
*Xiaobo Zhou, University of Texas Health Science Center, Houston, TX, USA,*
*Donald A. Adjeroh, West Virginia University (WVU), Morgantown, WV, USA.*

Long non-coding RNA (lncRNA) is arbitrarily defined as a transcript greater than 200 nucleotides (nt) with low or no potential to encode a protein. These lncRNA genes may reside in regions of the genome between protein-coding genes, but they may also overlap protein-coding regions, which results in several lncRNA biotypes, including intergenic, antisense, and intronic lncRNAs. According to GENCODE version 44 (v44) website statistics (www.gencodegenes.org/human/stats.html (accessed on 28 September 2023)), 31.8% (19,922/62,700) of the total genes annotated are lncRNA genes, while 23.0% (58,246/252,835) of the annotated transcripts are lncRNA loci transcripts. In contrast, just a tiny fraction of these is experimentally or manually curated and available and, in general, this is typically obtained from model species. These observations can tremendously impact lncRNA research. For that, we provide some discussion that may help researchers reflect on the current challenges in long non-coding RNA research. We point out open challenges crucial for the mathematical and computational modeling and analysis of lncRNAs and their roles in the cell. Some of these challenges require immediate attention, while others may require longer-term research to address. All the same, the goal is to raise the curiosity of the lncRNA community about these problems and to call attention to potential directions toward their resolution. We organized our discussion on the computational challenges into three broad groups: RNA biology, data challenges, and machine learning in lncRNA analysis.

### 2.1. RNA Biology: LncRNAs and Their Functions, ORFs, and Micropeptides

**Figure 1:**
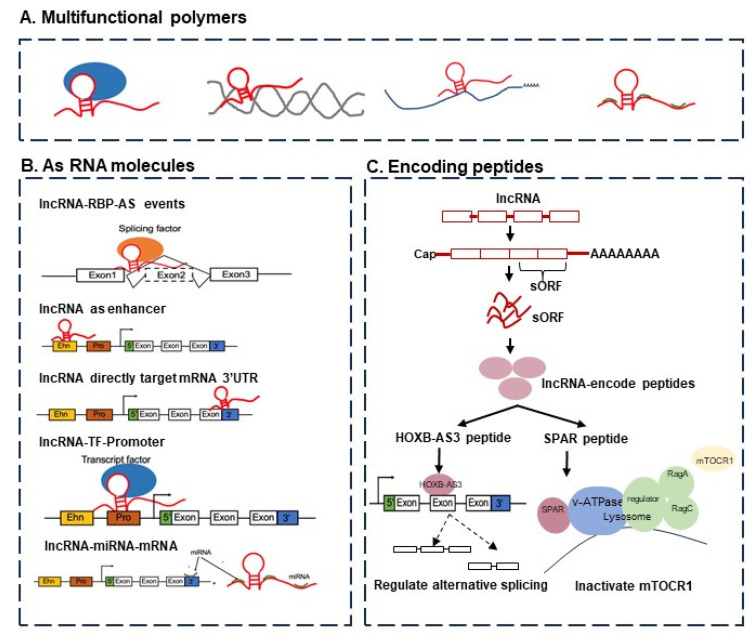
The functions of lncRNAs can be considered from two perspectives, namely, (i) from the viewpoint of RNA function alone and (ii) in terms of encoding peptides or proteins. (**A**) The multifunctional molecular complexes containing lncRNAs, including lncRNA–protein, lncRNA–DNA, lncRNA–mRNA, and lncRNA–miRNA. (**B**) The functions of lncRNAs as RNA molecules: lncRNAs regulate target genes by functioning as enhancers, lncRNAs regulate target genes by recruiting transcription factors, lncRNAs regulate alternative splicing events by interacting with splicing factors, and lncRNAs regulate mRNA by targeting the 3’-UTR region. (**C**) The functions of lncRNAs via encoding peptides or proteins able to regulate alternative splicing (e.g., HOXB-AS3 peptide) and gene expression (e.g., SPAR peptide).

Long non-coding RNAs are essential players in many cellular processes, from regular development to oncogenic transformation. Figure 1 provides a schematic view of the functions of lncRNAs. The rapid growth of genome-wide translation profiling and ribosome profiling has revealed that a number of small open reading frames (sORFs) within ncRNAs actually have peptide- or protein-coding potential [1,2]. The peptides or short proteins encoded by transcripts annotated as lncRNAs have been shown to be critical players in cancer development and progression. For example, a conserved 53 aa peptide encoded by the putative lncRNA HOXB-AS3 suppresses colon cancer growth by regulating the alternative splicing of pyruvate kinase M (PKM) and tumor metabolic reprogramming [1,3]. FBXW7-185aa is encoded by the circRNA FBXW7 and inhibits glioma growth [4]. Moreover, miPEP-200a and miPEP-200b are encoded by miR-200a and miR-200b, and respectively, inhibit the migration of prostate cancer cells via the suppression of the process of epithelial-to-mesenchymal transition [5].

**The addiction to ORF annotation in “non-coding” biology.** A recent study discussed the feature extraction approaches for biological sequences using different mathematical formulations [6]. In particular, the paper provided detailed mapping of the timeline on various tools for lncRNA identification (see Figure 1 in [6]). Of these tools, only 3 out of 21 (14.2%) did not use open reading frame (ORF) predictions or annotations in their methods. The conclusion was that most current approaches to lncRNA analysis are, paradoxically, still highly dependent on ORF information. How would such tools that highly depend on ORF information handle lncRNAs without ORF information? With the rise in awareness of the frequent incidence of small ORFs (sORFs or smORFs) in lncRNAs, what will the behavior of these tools be? How do they and how should they handle sORFs inside lncRNA genes? These are some open questions that the field will face imminently. One essential suggestion could be to consider alignment-free methods, using tools such as BASiNET [7], an alignment-free tool that uses network-based features to analyze biological sequences.

**Coding or non-coding RNA: What is a gene?** The definition of a gene has been substantially updated since the Central Dogma of Molecular Biology proposed by Watson and Crick. Researchers of the first post-genomic century have been deliberating over the increasingly complex question of what are coding and non-coding genes. This scenario brings one particular challenge, namely, when is the gene actually coding for a protein? For example, if a gene has 12 isoforms, of which some are coding and some are non-coding transcripts, should it be considered a coding or a non-coding gene? This is the case for the gene KCNK4-TEX40 (ID: ENSG00000257069), which has six transcripts annotated as protein-coding (including one with its CDS not defined), four annotated as “RNA with retained introns”, one as a product of nonsense-mediated decay, and a last one as an lncRNA. The question is the following: Do the isoforms not matter? This issue is not trivial, and also relates to the problem of bifunctional RNAs [8,9,10] where certain RNAs biologically exhibit both a protein-coding capacity and non-coding functional properties, as well as to complex transcriptional units such as SRA1, where a combination of alternative splicing and promoter choice lead to the production of distinct coding and non-coding transcripts, with different functions, from a single gene locus.

**Small in size, but a bigger challenge.** On a related challenge, there have been discussions about small, short, or micropeptides, or small ORFs. These peptides are derived from a myriad of genomic locations including coding, non-coding, intergenic, and untranslated regions (UTRs) with upstream or downstream coding gene regions, unveiling the remarkable complexity of cells [11]. Principally, the identification of lncRNA-encoded small peptides confronts the basic molecular biological concepts and brings up the question of whether or not these micropeptides are just noise. There are indications that some of these may not be just noise. A small peptide of 53 aa encoded by the lncRNA HOXB-AS3 binds to an mRNA and inhibits the formation of PKM2 to induce a tumor-suppressive effect [12] (see also Figure 1).

### 2.2. The Data Challenge in LncRNA Research: Datasets, Availability, Beyond Human Data 

**Datasets.** In the era of data-hungry machine learning and other artificial intelligence (AI) techniques, data have become a premium in successful applications of these approaches in a given domain. Not surprisingly, the availability of data and datasets has also become an urgent challenge in the computational analysis of lncRNAs and their functions. Several datasets have become available on lncRNAs. For instance, RNALocate v2.0 [13] contains RNA subcellular localization entries validated by experimental evidence. The database contains information on lncRNAs located in the nucleus, cytoplasm, ribosome, exosome, nucleoplasm, chromatin, cytosol, endoplasmic reticulum, and plasma membrane. LncATLAS [14], a key database for the subcellular localization of lncRNAs, provides a cytoplasmic/nuclear relative concentration index (CN-RCI), derived from GENCODE (Ensembl) RNA-Seq measurements for lncRNAs and mRNAs across 15 cell lines.

From the emerging importance of ncRNAs in cancer, studying regulations related to ncRNAs and their coding potential is essential to fully understanding cancer progression and identifying therapeutic targets. Several databases are now available on cancer-related studies providing annotations for interaction and ncRNA–disease associations. Examples of these include RNAcentral [15], NONCODE [16], NoncoRNA [17], NPInter [18], ViRBase [19], MNDR [20], EVAtlas [21], ncRNA-eQTL [22], and NSDNA [23]. Some datasets such as ncEP [24], FuncPEP [25], and SPENCER [26] only contain experimentally validated and functionally characterized ncRNA-encoded peptides. lncRNAfunc [27] is a newly developed knowledgebase focusing on the annotation of lncRNA function in human cancer. TransLnc [28] provides the potential peptides encoded by lncRNAs, but this database only includes lncRNAs and lacks circRNAs and miRNAs, which are also required to fully understand ncRNAs’ coding potential of action in cancer. Recently, a manually curated dataset of bifunctional RNAs was introduced [10].

To date, systematic annotations of coding potentials for ncRNAs have been unavailable. There is also an urgent need to develop a comprehensive resource for translatable ncRNAs that extends neoantigens for investigating the translation capacity of ncRNAs and for expanding investigations on the cancer immunopeptidome. Similar comprehensive resources are also required for applications of lncRNAs and other ncRNAs outside of cancer. But other challenges still abound.

**Benchmarking, standardized nomenclature, and discrepancies.** As noted by the creators of the Extensive de novo TE Annotator (EDTA) in [29], there is a significant lack of benchmarking and standardized studies to help to organize the diversity of studies and datasets in lncRNA research. We have the HUGO Gene Nomenclature Committee [30] and similar reports [31,32], for example. All these initiatives are crucial for biological research; however, they have focused mainly on humans. Science is much more than the human genome. In plants, there are disparate lncRNA datasets, with differences in their annotations. A case in point is the difference in lncRNA annotations between two popular datasets CANTATAdb [33,34] and GreeNC [35,36]. What is the overlap among these available databases? The lack of experimental data raises the need for a careful analysis of potential discrepancies and to possibly arrive at a consensus across different datasets.

**Big data age does not always mean good data availability.** By 2019, there were over 230 databases dedicated only to ncRNAs [37,38]. For miRNA, the most studied ncRNA, only about 13% of the databases made their FASTA sequences or similar files available, and for lncRNAs, it was even a tinier fraction [37,38]. The scientific community has to wake up to these unbelievable statistics because information is needed to build novel and cutting-edge methods. Having access to data is fundamental to improving in silico research. This seems obvious, but it is not always the case. This challenge brings one more reflection that less is actually more. From a supervised machine learning point of view, a higher quality but smaller number of experimentally validated data samples is perhaps better than big data without validated data or curated labels. We also do concede that, when one considers certain machine learning models, such as self-supervised learning, large unannotated datasets can still be valuable for learning important representations from the data. A related challenge is about how the scientific community makes data available. This is perhaps a more general problem, beyond lncRNAs and genomics research. There are cases where a dataset was published but not updated; even worse, the dataset may have been discontinued and made no longer available, without providing access to the old versions of the dataset. This has been observed even for datasets published as part of some papers in relatively high-quality (Q1) journals. Another aspect is that, oftentimes, data are available as supplementary material and are often incomplete, for instance, without complete sequence information or other relevant attributes. These data need to be easier to obtain. Making data available in several formats (e.g., FASTA, GFF, BED, TSV), using an uncomplicated download protocol is the way to go. Some journals and conferences are already moving toward this requirement.

**Beyond lncRNA data from humans and other model organisms.** The pandemic created an awareness and significantly increased our consciousness with respect to the importance of using data from non-model species in important biomedical investigations. Whether such data are coming from a bat or from a pangolin does not matter. What really matters is that only focusing on humans or mammals limits our analysis and perspective to just a tiny fraction of biodiversity. Only by deeply exploring other species can we prepare ourselves for future pandemics and novel drug development. A well-explored model plant is *Arabidopsis thaliana*, whose genome, for example, is quite comparatively different from the coffee genome. For lncRNAs, and for ncRNAs or other types of genomic research for that matter, there is an urgent need to consider information and genomic data from other species, beyond the human genome, or the standard model species.

### 2.3. Modern Machine Learning in LncRNA Research: Deep Learning and LLMs

We consider there to be two problems in the application of artificial intelligence (AI) techniques in lncRNA analysis, specifically the use of deep learning models in lncRNA localization and the use of large language models (LLMs) in studying lncRNAs.

**LncRNA subcellular localization: More challenging than it appears.** There has been vigorous research on computational approaches to the problem of lncRNA identification [39,40,41] and on the issue of mRNA localization [42,43,44,45]. LncRNA subcellular localization has also attracted some recent attention [46], and various machine learning methods have been proposed [47,48,49]. Some of these methods reported relatively high performance, for instance, relatively high accuracy, and in some cases, above 90%. However, a closer analysis showed that most of these methods had problems in their data, for instance, working with significantly imbalanced data, where the machine learning model was basically memorizing the majority class. In some others, there was observed significant leakage in the data, whereby there was leakage of data from the training to the validation sets or to the test sets. In some other cases, some methods tended to directly or indirectly ignore or eliminate the difficult cases (for instance, based on the thresholds used to declare the localization, say nuclear versus cytoplasmic localization, for the case of binary localization). The result was that the true performance of these methods was typically in the 50% to 65% range (for binary nuclear versus cytoplasmic localization), generally much lower than some of the reported performance levels. However, the results for these methods demonstrated that the computational analysis of lncRNA localization, be it for binary class or multi-class localization, is still a critical challenge, especially using only sequence information. This thus calls for more careful benchmarking of existing machine learning methods for this problem. For fairness, such benchmarking will have to be performed on the same dataset across different methods and on the same or comparable hardware configurations. (This obviously relates to the challenge of the datasets introduced previously). Beyond benchmarking, there is also need for more specialized machine learning and deep learning techniques that can exploit the special nature and characteristics of lncRNAs, including information on their sequences, structures, conformations, and types. Clearly, this need for more sophisticated deep learning algorithms also applies to the general area of lncRNA and ncRNA analysis, and not just on lncRNA localization.

**Large Language Models (LLMs) for lncRNAs.** Current lncRNA detection methods are mainly designed based on transcriptome sequencing data. Language models such as Bidirectional Encoder Representations from Transformers (BERT) and Generative Pre-training (GPT) have received an extensive attention recently because of the good performance of the transformer involved. They have been applied in various directions and fields. Recently, scientists found that some sORFs were also derived from lncRNAs. sORF-encoded peptides (SEPs) with a length of less than 100 amino acids were found to be involved in cancer suppression and cellular metabolism. Feng et. al. developed LncCat [50], an ORF attention model for identifying lncRNAs using a natural language model, which showed an impressive performance. This model could be improved by pre-training and fine-tuning the BERT and GPT models. Other models such as the BART model [51] could be applied to this problem too. We could potentially identify a lot of more lncRNAs with these latest approaches. Along the lines of the improved techniques mentioned earlier, a similar case can also be made beyond methods for improved lncRNA identification, but for the case of developing specialized LLMs for general analysis and understanding lncRNAs and their functions.

### 2.4. Looking to the Future: Toward Improved Research and Applications

Two aspects are helping to provide better lncRNA annotation and, consequently, discover better lncRNA functional information. The first is developments in long-read sequencing technology which will help to sequence full-length transcripts and lncRNAs. The second is the deep sequencing of genomes (for example, the Genome 10K Project [52]; 100,000 Genomes— in Africa, for Africa [53]; 100,000 Genome Project in the UK [54]; and 10KP: 10,000 Plant Genomes Project [55]). These are examples of how the large-scale availability of samples in the same species could help to comprehend the diversity, conservation, and variability among lncRNAs. In the same way, single-cell sequencing will help to look at these data as individual cells, enabling cell-to-cell communication. Further, the increasing availability of information about chromatin packaging and accessibility, for instance, from assays for transposase-accessible chromatin with sequencing (ATAC-Seq), could also open up another vista for the improved computational analysis of lncRNAs. Combining insights from ATAC-Seq with information from RNA-Seq could lead to new breakthroughs in the machine understanding of the functions and activities of ncRNAs in general, and lncRNAs in particular. These technologies coupled with improvements and new developments in computational methods (both hardware (e.g., processor architectures) and software (e.g., AI)) will improve future research on lncRNAs and create new applications and possibilities like never before seen.

## 3. Challenges and Rewards of Modelling Cancer-Associated Long Non-Coding RNAs In Vivo

*Nadya Dimitrova, Yale University, New Haven, CT, USA*

Cancer initiation and progression to a deadly and metastatic disease is a multi-step process that involves the gradual acquisition of numerous genetic and epigenetic aberrations of cellular effectors, involved in proliferation, survival, differentiation, and cell-to-cell interactions [56]. The discovery that mammalian genomes encode thousands of long non-coding RNAs (lncRNAs) has spurred investigation into whether the non-coding genome contains an untapped reservoir of novel modulators or perhaps even novel effectors of cancer processes [57,58]. To date, the in vivo evidence that lncRNAs are themselves effectors in cancer is sparse. On the other hand, the potential for lncRNAs to act as modulators of key nodes in cancer pathways is steadily gathering experimental support, including in organismal models [59]. Here, we focus on how the dysregulation of lncRNAs can rewire cellular pathways, creating points of vulnerability exploited during cancer evolution. We then describe how genetically engineered mouse models (GEMMs) of cancer can be carefully harnessed to define the scope of lncRNAs’ contribution in cancer.

While lncRNAs are rarely mutated in cancer, comparative analyses of normal and tumoral tissues have revealed extensive cancer-linked alterations in lncRNA expression patterns [60]. Global gene expression analyses have highlighted numerous differentially expressed lncRNAs and, in many cases, robustly linked differential lncRNA expression patterns with cancer types and clinical outcomes. For example, the overexpression of the lncRNAs metastasis-associated lung adenocarcinoma transcript 1 (*Malat1*) and Hox transcript antisense RNA (*HOTAIR*) has been strongly associated with metastatic lung and breast adenocarcinomas [61,62]. In some cases, genomic analyses of non-coding regions have provided a genetic mechanism for altered lncRNA expression, such as the somatic copy number variations (SCNVs) in plasmacytoma variant translocation 1 (*PVT1*) [63] and focally amplified lncRNA 1 (*FAL1*) [64] loci and the single nucleotide polymorphisms (SNPs) detected in enhancer and promoter regions of prostate cancer-associated transcript 1 (*PCAT1*) [65] and neuroblastoma-associated transcript 1 (*NBAT1*) [66], respectively. The contribution of dysregulated lncRNA expression to cancer has been an important ongoing question to pursue.

Current models propose that lncRNAs act primarily through two mechanisms: *cis*-regulatory, acting as local activators or repressors of the expression of neighboring genes, and *trans*-regulatory, acting as global modulators of cellular processes throughout the nucleus and in the cytoplasm [67]. The subcellular localization of an lncRNA detected by single-molecule RNA fluorescence in situ hybridization (smRNA-FISH) can provide clues about its mechanism. Many *cis*-regulatory lncRNAs, including *PVT1*, accumulate at their site of transcription [68], while *trans*-regulatory lncRNAs, such as *Malat1*, tend to have a wider cellular distribution [69]. When designing gain-of-function and loss-of-function models to elucidate lncRNA function, it has been essential to consider the mechanism involved [70]. For example, the functions of *cis*-regulatory lncRNAs cannot be reliably studied using RNAi- or ASO-based knockdown approaches and their activities cannot be recapitulated with exogenously expressed constructs. In addition, genetic modifications of *cis*-regulatory lncRNA loci have to be assessed for their RNA-independent effects through perturbations of transcriptional and DNA regulatory elements. On the other hand, lncRNA/target accessibility and relative abundance have to be incorporated in models for *trans*-regulatory lncRNAs.

When one considers these caveats, it becomes apparent that designing experiments to study the contribution of dysregulated lncRNA expression in cancer, especially at the organismal level, can be very challenging. Efforts to investigate two well-studied cancer-associated lncRNAs, *PVT1* and *Malat1*, described below, illustrate the discordant results from alternative approaches and highlight the importance of carefully designed studies to dissect the contributions of lncRNAs in cancer initiation and progression.

Frequently amplified in cancer, *PVT1* offers a striking example of the challenges encountered when studying cancer lncRNAs. Initial work supported the proposed oncogenic function of *PVT1* in a murine model of breast cancer where genetically engineered *Myc-Pvt1* co-amplification was observed to be more tumorigenic than *Myc* amplification alone [71]. However, subsequent studies discovered tumor-suppressive elements in the *Pvt1* locus, including a DNA boundary element [72] and a p53-regulated isoform, *Pvt1b*, which acted *in cis* to downregulate *Myc* expression in response to genotoxic and oncogenic stress and during cellular senescence [68]. Inhibition of the p53-activated isoform *Pvt1b* by CRISPR mutagenesis of the p53 binding element in vivo led to larger tumors in an autochthonous mouse model of lung adenocarcinoma [68]. This finding placed *Pvt1b* downstream of the p53 tumor-suppressive pathway and indicated a specific role for *Pvt1b* in restraining growth early in tumor development through *Myc* repression. Thus, there is exciting potential for using the *Pvt1* locus as a therapeutic handle to modulating *Myc* levels in cancer. However, additional carefully designed GEMMs are needed to deconvolve the oncogenic and tumor-suppressive elements in the *Pvt1* locus and to dissect the interplay between regulatory DNA and RNA elements.

Another example of the biological and technical complexities associated with studying the roles of lncRNAs in cancer is provided by *Malat1*, whose overexpression is strongly associated with metastatic disease and poor patient prognosis across a remarkably wide range of cancer types [73]. Despite its ubiquitous expression, strong conservation, and high abundance in all mammalian cells, both the normal and the cancer functions of *Malat1* have remained a mystery. One of the main caveats of previous studies has been the use of three independent *Malat1* knockout mouse models, which have led to conflicting results likely because they did not properly recapitulate the potential gain-of-function or dominant phenotypes that *Malat1* overexpression may have imparted in the context of cancer [74,75]. Indeed, in unpublished work from our lab, CRISPR activation-guided epigenetic overexpression of endogenous *Malat1* in an autochthonous mouse model of lung adenocarcinoma confirmed that increased *Malat1* expression is a driver of cancer progression. We found that while *Malat1* does not modulate cellular proliferation, it cooperates with p53 loss to reprogram the tumor microenvironment and promote metastatic disease. The data indicated that *Malat1* drives tumor progression, rather than tumor initiation, consistent with its association with advanced disease and poor prognosis in patients. One of the outstanding questions is the link or lack thereof between *Malat1*’s normal and oncogenic functions and mechanisms. Another important area for further investigation is how targeting *Malat1* or its downstream targets may be harnessed to limit metastatic disease.

Attempting to model the dysregulation of lncRNAs in cancer models has not been without its challenges. However, growing efforts that take into account the evolving functions and mechanisms of lncRNAs also reflect the important insights that can be gained from well-designed studies.

## 4. The Human Y Chromosome, Long Non-Coding RNAs, and Cancer: Challenges and Opportunities

*Ivan Martinez, West Virginia University, Morgantown, WV, USA*

From amongst the 23 pairs of chromosomes comprising the human genome, the human Y chromosome is the smallest, containing only a little over 62 million base pairs. (Chromosome 1, the largest, spans about 249 million base pairs.) [76] For that reason, it is not surprising that the Y chromosome harbors, and expresses, fewer protein-coding genes than any other chromosome in the human genome [77]. Originally, the X and Y chromosomes contained the same amount of genetic information, but during evolution, the Y chromosome lost most of its genes, while maintaining the sex-determining SRY gene. Although the reference assembly of the human genome, building upon the Human Genome Project’s original sequence completed in 2003, was updated in 2013, and again with supplementary annotations in 2019, named GRCh38 [78], the complete sequencing of the human Y chromosome was not achieved until 2023 [76]. The main difficulty in sequencing the Y chromosome was engendered by its high amount of complex repetitive DNA structures, such as long palindromes, tandem repeats, and segmental duplications. This property is unique to the Y chromosome. There are other difficulties in the study of the human Y chromosome: in particular, during the process of aging, men’s cells start to lose their Y chromosome [79], a condition that can be exacerbated by smoking [80]. The occasional loss of the Y chromosome in different male cell types is known as mosaic loss of Y chromosome (mLoY). This uneven mLoY has been linked to several diseases, such as a higher incidence of Alzheimer’s disease [81], cardiac fibrosis [82], and deficient immune response [83], and a higher risk of cancer development [84]. A recent study using a pan-cancer approach revealed that different types of male cancers have different amounts of mLoY [85]. The study suggested that mLoY is a reflection of genetic instability in cancer cells and could be associated with a higher incidence of mutation rates [85]. All these findings pose an interesting question: How could the loss of a small chromosome, such as the Y chromosome, which only contains a few protein-coding genes, be so important in cancer development and aggressiveness? The answer may be found in the different types of non-coding RNA (ncRNA) transcripts expressed from the Y chromosome.

In comparison to other ncRNAs, such as microRNAs, small nuclear RNAs, and other “classical” ncRNA types, long non-coding RNAs (lncRNAs) have a higher genetic and functional diversity. In general, lncRNAs demonstrate a low rate of gene expression in normal cells, relative to the typical expression levels of protein-coding mRNAs, but lncRNA expression is altered in a wide variety of human cancers [86]. Interestingly, most lncRNAs, instead of having a high extent of evolutionary sequence conservation comparable to the majority of protein-coding genes, rely more on secondary structure conservation, which affords them a higher level of functional versatility [87]. Previously, it was accepted that lncRNAs, by definition, were unable to be translated into proteins, as they were assumed to lack bona fide open reading frames (ORFs). However, recent studies, starting with the findings from the Lipovich laboratory within the framework of Phase II of the ENCODE Consortium, experimentally showed, using direct methods such as mass spectrometry and indirect methods such as ribosome profiling, that certain lncRNAs have short ORFs and are able to be translated into “micropeptides” with a median size of 44 amino acids [88,89], even though these ORFs are distinct—in terms of their micro size, (recent) evolution, and (lack of) any database homologies or predicted functions—from conventional mRNA ORFs, indicating that despite containing short ORFs, these lncRNAs should not be reclassified as protein-coding. In general, lncRNAs have been classified into four major groups: antisense to protein-coding genes, sense-overlapping, intergenic, and intronic [90]. Antisense lncRNAs are transcribed from the antisense DNA strand of a protein-coding “sense” gene, and in contemporary nomenclature, they are commonly named with the protein-coding gene name with the addition of an -AS (antisense) at the end [91], for instance, FAM83H-AS1, AFAP1-AS1, and ASMTL-AS1. Sense lncRNAs are encoded on the same DNA strand as the gene they have been named after even though their sequence includes some of the gene’s exonic regions which may not be translated (because they reside in untranslated regions or because the of isoform dependence of the reading frame), as is the case with the lncRNA DAPLAR [92]. Intergenic lncRNAs, also known as long intergenic non-coding RNAs (lincRNAs), are the most common lncRNA type. These lncRNAs do not overlap any known coding genes [93], and include the linc-SPRY3 family [94]. Intronic lncRNAs, as the name implies, are expressed within the intronic regions of protein-coding genes [95]. All these types of lncRNAs are transcribed from the human Y chromosome, but intergenic lncRNAs are the most commonly found there—perhaps due to the paucity of annotated protein-coding genes on this chromosome. Until now, the expression of more than 50 lncRNAs from the human Y chromosome has been documented, and evidence is emerging from recent studies that some of these ncRNAs are contributing to the etiology and pathogenesis of several human diseases.

A global gene expression analysis of male human brain embryos has unraveled six new lncRNAs (KDM5D, TTTY14, UTY, TTTY15, ARSEP1, and TXLNG2P) expressed from the Y chromosome that could be potentially involved in central nervous system development [96]. Dysregulation of these lncRNAs and others plays a role in many diseases including cancer. For example, lnc-KDM5D-4 has a role in fatty liver formation and cellular inflammation associated with atherosclerosis and coronary artery disease in men [97]. Also, the expression of the Y chromosome lncRNA TTTY15 was found to be frequently elevated in prostate cancer tissue, in comparison to normal tissue, proving that this lncRNA is not a mere biomarker but a functional contributor to cell proliferation; repression of this lncRNA showed a tumor-suppressive effect [98]. In multiple myeloma, the antisense lncRNA ZFY-AS1 was found to be a protective prognostic biomarker through bioinformatics analysis [99]. Another example is LINC00278, an lncRNA able to be translated into a small peptide (micropeptide), known as Yin Yang 1, that is important in the development of esophageal carcinomas [100]. Interestingly, LINC00278 was found to inhibit the growth of laryngeal squamous cell carcinoma cells in in vitro and in vivo models, by negatively regulating the AKT/mTOR signaling pathway via the downregulation of COL4A1/COL4A2 [101].

Our group discovered a family of lncRNAs expressed from the Y chromosome that directly contribute to radiation therapy susceptibility in male non-small-cell lung carcinomas (NSCLCs) [94]. We found three lncRNAs named lnc-SPRY3 RNAs (also known as lnc-BPY2C), which exhibited dose-dependent expression after radiation in radiosensitive male NSCLC cell lines, but not in radioresistant male NSCLC cell lines. Cytogenetic analysis revealed that the radioresistant male NSCLC cell lines lost their Y chromosome (mLoY), and consequently, lnc-SPRY3 RNAs did not exist in those cell lines. Gain- and loss-of-function experiments confirmed that the lnc-SPRY3 family functionally contributes to the radiation susceptibility of male NSCLC cells. More recent unpublished findings from our group have highlighted that the exogenous expression of the lnc-SPRY3 RNAs in female NSCLC cells, as well as mouse NSCLC cells, which also induced radiation susceptibility, suggesting that not only are the pathways affected by these lncRNAs present in female cells and other mammalian cells, but also that a potential use of these lncRNAs as therapeutic molecules may be feasible. The advantage of using mouse NSCLC cells expressing these human lncRNAs is enormous, because we can ask questions about the tumor microenvironment and the immune response, as well as address the functional impact of the differences between the mouse and human Y chromosomes, in well-established in vivo mouse xenograft models of cancer. One of the greatest obstacles of using mouse models to study lncRNAs expressed from the Y chromosome is the vast difference between the human and the mouse Y chromosomes. For example, the mouse Y chromosome is 99.9% euchromatin, expressing almost 700 protein-coding genes [102]. In contrast, the human Y chromosome is largely heterochromatic and has less than 40% euchromatin [76], serving as an example of the extensive, and often underappreciated, human–mouse differences in the non-coding sequence space of the genome. In this context, it is important to remember that, unlike protein-coding genes, most lncRNAs are not conserved between mammalian lineages, and hence, most human lncRNAs appear to be primate-specific in evolution, based on conventional nucleotide sequence homology analyses and multispecies alignments. Reflecting this trend, the linc-SPRY3 lncRNA genes are only present in humans and some nonhuman primates and are absent in mice.

An orthogonal opportunity to understand the importance of Y chromosome-encoded lncRNAs in cancer is afforded by the increasing adoption of artificial intelligence (AI) in bioinformatics. There is emerging evidence that AI can predict chromosomal aneuploidies in glioblastomas, using an imaging-based fully automated method [103]. Furthermore, AI was used to review histopathological images of colorectal cancer tumors to predict DNA yields [104]. Our group is interested in using AI to identify and characterize specific histopathological features found in male NSCLC tumors with the loss of the Y chromosome, in order to correlate these images with tumor staging, overall survival, and smoking history. By using DNA-FISH and hematoxylin and eosin (H&E) stained images from male NSCLC tissue arrays, we are planning to test different segmentation and convolutional neuronal network algorithms to identify differences in histopathological features (including cell size, nuclear cluster, and nuclear texture) that predict the loss of the Y chromosome.

Summarily, here we highlight that the study of lncRNAs expressed from the human Y chromosome is an uncharted new field with exceptionally high potential to generate new cancer-related findings that could explain in part the sex-specific differences in cancer treatment efficacy as well as differences in overall survival between male and female cancer patients, simultaneously providing the mechanistic insights that could guide the development of lncRNA-driven therapeutics in NSCLC and beyond.

## 5. Non-Coding RNAs in the Genetics of Sports

*Ekaterina G. Derevyanchuk, Southern Federal University, Rostov-on-Don, Russian Federation. Leonard Lipovich, Shenzhen Huayuan Biological Science Research Institute, Shenzhen, China, and School of Medicine, Wayne State University, Detroit, Michigan, USA.*

To date, over 200 genes have been associated with the development and manifestation of human traits related to physical activity, exercise, and endurance [105,106]. The majority of these genes were annotated by Bouchard and colleagues in a human genetic map of physical activity [107,108]. A detailed study of these genes is required in order to understand how they convey the underlying phenotypes, and hence, how to properly design a personalized sports training regimen that accounts for genetics, accurately predicts individual athletes’ capabilities, proactively identifies exercise-related health problems, and facilitates the detection of the new threat of gene-based doping. In the past two decades, the progressive emergence of high-throughput technologies to monitor transcriptome profiling has revealed that, while the exons of protein-coding genes account for a mere 1.5% of the 3,300,000,000-base human genome, more than 90% of the human genome is transcribed into non-coding RNA (ncRNA) transcripts. The most abundant class of these long non-coding RNAs (lncRNAs), encoded by 40,000 of the estimated 60,000 human genes, has been shown to be essential for a wide range of fundamental cellular and biological processes in health and disease [109,110,111].

Motivated by these revelations, our studies have been focused on the identification and localization of miRNA binding sites in the 3′UTRs of, and lncRNA transcripts adjoining and overlapping (including antisense and bidirectionally promoted transcripts of), key genes previously shown to be among the determinants of endurance and speed strength qualities (*PPARD*, *NRF2*, *PGC-1*, *HIF-1*, *HIF-2*, *GYS1*, *HBA1*, *HBA2*, *HBB*, *ADRB2*, *NOS3*, *CHRM2*, *UCP2*, *UCP3*, *VEGF*, and *EPO*), genes for muscle work efficiency (*ACE*, *CK-MM*, *ACTN3*, *MLCK*, *AMPD1*, and *IGF-1*), and genes responsible for psychological characteristics relevant to exercise (*5HTT*, *BDNF*—already known to be regulated by the antisense lncRNA *BDNF-AS1*—as discussed in the Human Brain Activity section of this mini-review, *HTR2A (SR)*, *DRD2*, and *MTHFR*).

We used the TargetScan Human 8.0 resource to identify microRNA binding sites inside the studied genes (http://www.targetscan.org/vert_80/ (accessed on 5 October 2021)). TargetScan predicts the biological targets of miRNAs at protein-coding mRNA 3′UTRs by searching for the presence of conserved 8mer and 7mer sites that match the seed region of each miRNA. Sites are identified with mismatches in the seed region that are compensated by conserved 3’ pairing. We used a Pct cutoff = 0.8 to select only significant miRNA binding sites. We identified binding sites for 26 microRNAs inside the studied genes.

Furthermore, we performed a search for disease-related SNPs in miRNA binding sites. We used miRdSNP, a database of disease-associated SNPs in miRNA target sites on 3’UTRs of human genes (http://mirdsnp.ccr.buffalo.edu/, (accessed on 28 September 2023)). We identified 21 miRNAs that were literature-supported (PubMed) disease-associated SNPs at their target sites in our selected set of genes. This study needs to be complemented in the future by competing endogenous RNA (ceRNA) prediction tools that may help identify, for example, miRNA–lncRNA interactions that sponge miRNAs away from important mRNA targets in the endurance and physical ability contexts.

Although, in addition to miRNAs, lncRNAs play critical roles in various biological functions and disease processes, human physical qualities related to lncRNAs have rarely been investigated to date. We carried out bioinformatics analysis using the UCSC Genome Browser and its underlying UCSC Genome Database (https://genome.ucsc.edu/, (accessed on 23 May 2023)) to find lncRNAs transcribed near, or overlapping, selected genes. We analyzed the length and tissue expression distribution of the candidate lncRNA identified, which included several TCONS transcripts (transcripts of uncertain coding potential, contributed to databases by high-throughput transcriptome projects but lacking detailed laboratory-based annotations), and we found that these characteristics vary widely. In particular, overall, we observed a lack of their expression in muscle tissue, except the HELLPAR lncRNA (a cause of HELLP Syndrome, a complication of pregnancy), supported by the TCONS_l2_00006386, TCONS_l2_00006387, and TCONS_l2_00006388 transcripts located downstream of the *IGF1* gene and, intriguingly, expressed mainly in skeletal muscle, according to the “LincRNA RNA-seq” UCSC Genome Browser dataset.

It was already evident from the literature and from our pilot analysis that both short and long non-coding RNAs had great potential for further studies in the nascent field of sports genomics. The obtained findings should promote the understanding of possible lncRNA functions in the regulation of human physical qualities, with implications that range well beyond extreme sports such as deep-sea diving and high-altitude alpinism, in particular for the transcriptome-based control of endurance and extreme physiology, in situations such as recovery from natural and manmade disasters.

## 6. Long Non-Coding RNAs in COVID-19

*Tatiana P. Shkurat, Southern Federal University, Rostov-on-Don, Russian Federation. Leonard Lipovich, Shenzhen Huayuan Biological Science Research Institute, Shenzhen, China, and School of Medicine, Wayne State University, Detroit, Michigan, USA.*

During the opening decades of the post-genomic era, it has become increasingly clear that the majority of significant disease-associated variants discovered in Genome-Wide Association Studies (GWAS) across all human diseases reside in non-coding regions of the genome [112], and at the same time, long non-coding RNAs (lncRNAs) have been emerging as drivers of different diseases [113]. Tens of thousands of lncRNAs genes regulate the activity of protein-coding genes in various tissues and their response to environmental influences. Nevertheless, most lncRNAs’ disease contributions and druggability potential remain poorly understood. To date (23 May 2023), more than 6.9 million people on our planet have died from the SARS-CoV-2 virus and more than 765.9 million have been infected (https://covid19.who.int, (accessed on 23 May 2023)). Extraordinary worldwide research and clinical efforts have been undertaken to understand the complex mechanisms of SARS-CoV-2 infection.

Coronaviruses that cause disease in humans have been transmitted to humans from animals and to domestic animals from bats. Viral mutations occur in animal hosts. Current consensus converges on the conclusion that, during passage through animal hosts, the virus accumulated mutations in the spike protein gene. This spike is important for the docking of SARS-CoV-2 to human cells. Comparative analysis of SARS-CoV-2 with other coronaviruses showed that this virus has a stretch of six new amino acids in its spike protein, Tyr–Leu–Thr–Pro–Gly–Asp [114]. New mutations increase dramatically in the SARS-CoV-2 spike protein when the virus is transmitted person to person, as the virus mutates mainly when it replicates in host cells. Analysis of 48635 SARS-CoV-2 genomes highlighted a total of 353341 mutation events, relative to the NC_045512.2 Wuhan reference genome, with an average of 7.2 mutations per sample [115]. Frequently occurring variants have been found in both the non-coding and the protein-coding regions of the viral genome. But the severity of the disease does not depend solely on the characteristics of the virus. The clinical manifestations of COVID-19 are extraordinarily variable and range from a complete absence of symptoms to severe respiratory failure and death. This extreme and unusual clinical variability suggests that host genetics (i.e., genomic variants) play a strong role in the susceptibility to symptomatic disease and in and impact of the wide spectrum of manifestations of COVID-19.

We meta-analyzed data from GWAS and other host-side genetic studies of COVID, noticing the high diversity of the datasets. Nine causal SNPs and eleven pathogenic genes in COVID-19 have been identified in populations in Spain and Italy [116]. In the Chinese population, SNP variants common with other populations (including in the *ABO* blood group gene, previously implicated in susceptibility to diabetes and metabolic disease and harboring an antisense lncRNA that overlaps its first exon (L. Lipovich and E.L. Kleinrbink, pers. comm., unpublished), and in the antisense lncRNA *FOXP4-AS1*) were found to be significantly associated with COVID-19 severity. The rare risk variant in *MEF2B*, on the other hand, is specific to East Asian populations and confers an approximately eightfold increase in the risk of severe COVID-19 among carriers [117]. A meta-analysis of GWAS from more than 125,000 cases in 60 studies from 25 countries showed that genes at several loci, including *SFTPD*, *MUC5B*, and *ACE2*, contained variants that could be confidently used to predict the susceptibility to, and the severity of, symptomatic disease [118].

Intriguingly, several studies have highlighted that the expression of specific antisense lncRNAs was deregulated in patients with COVID-19 and correlated with the severity of COVID-19, and that this may play a role in the pathogenesis of this disease. Reduced expression of the lncRNAs *A2M-AS1* and *FLVCR1*, and increased expression of the lncRNAs *DBH-AS1*, *FLVCR1-DT*, and *NCBP2AS2*, was observed in patients with COVID-19. Both *FLVCR1-DT* and *NCBP2AS2* showed a positive correlation with interleukin-6 (IL-6). *DBH-AS1* and *FLVCR1-DT* had a significant association with mortality, complications, and mechanical ventilation. A significant negative correlation was found between *A2M-AS1* and *NCBP2AS2-1* as well as between *FLVCR1* and *FLVCR1-DT* [119], the latter consistent with many findings of antisense lncRNAs negatively regulating their cognate-sense mRNAs transcribed from the same locus, mentioned in other sections of this mini-review. In another recent study [120], it was shown that, in the severe form of COVID-19, 898 differentially expressed lncRNAs were detected in the peripheral blood of patients: 414 upregulated and 484 downregulated. In that study, through GO and KEGG gene ontology and functional category enrichment analysis, the authors reported the detected lncRNAs to have previously known functions in neurons, lung cancer, and organ injury.

We conducted a bioinformatics analysis of the localization of human long non-coding RNAs genes located within 50 kb of each significant phenotype-associated genetic variant SNP mapped in COVID GWAS and virus–host interactions. It is interesting to note that the majority of these SNPs are located on chromosome 2. In comparison, on chromosomes 6 (heavily involved in immunity thanks to hosting the MHC complex genes) and 19, only very small numbers of significant SNPs are located on similarly sized genomic fragments. We used data from the COVID GWAS v4 (genome-euro.ucsc.edu) and COVID19-hg GWAS round 5 meta-analyses (www.covid19hg.org). We examined all SNPs reported by these efforts for possible localization within lncRNA genes. We then constructed maps of interactions for the identified COVID severity, SNP-containing lncRNAs (IL10RB-AS1, MGC57346, CCR5AS) with miRNA and protein-coding genes. Using the LncRRIsearch web server (http://rtools.cbrc.jp/LncRRIsearch/, (accessed on 23 May 2023)), we predicted interactions between the miRNAs known to target the highlighted genes and various lncRNAs. Then, we analyzed the significant SNPs in the protein-coding genes targeted by miRNAs in this context and identified all lncRNAs that had the potential to interact with them. For example, we identified three lncRNAs that showed the highest probability of interaction with the tyrosine kinase (*TYK2*) gene: RP11-573D15.8-01 (antisense to the FETUB/fetuin-B protease inhibitor gene), AP006621.9-001 (antisense to the EPS8L2 gene involved in actin cytoskeleton organization), and RP11-95O2.5-001 (antisense to CELF4). Each of these lncRNAs also interacts with more than 15 different miRNAs. This prototype points to the possibility of a massive and unbiased analysis of how, specifically, lncRNAs regulate coding gene activity, and of their relationship with miRNAs and with each other. These studies will empower quantitative assessments of the viral infection phenotypic response range of any given host genotype, hence heralding the implementation of genome-driven, RNA-empowered, personalized medicine for more effective responses to current and future pandemics.

## 7. Deciphering Roles for LncRNAs in Human Brain Activity, Disease, and Death

*Jeffrey A. Loeb, Department of Neurology and Rehabilitation, University of Illinois at Chicago, Chicago, USA*

The human brain has evolved to generate extraordinary capabilities enabling complex behaviors not present in other species. Due to their often recent evolutionary origins relative to older conserved protein-coding genes, as well as to their more rapid rate of evolution as a consequence of being unencumbered by protein-coding open reading frames, lncRNAs have a significant potential to contribute to recent-origin and species-specific traits, including the phenotypic uniqueness of the human brain, through a wide variety of functions that are still poorly understood. At the same time, and perhaps because of these capabilities, the human brain suffers from a number of diseases related to both too much brain activity (epilepsy) and too little (neurodegeneration). Given these strong evolutionary differences, a major challenge in deciphering the roles of lncRNAs in human brain function and disease comes from a critical need to study them in the human brain rather than in other species.

For most other human brain diseases including Alzheimer’s disease, we have to wait until death to collect tissues for research. When comparing the genomic landscape between fresh human brain samples and postmortem samples, we discovered marked differences in coding and non-coding gene expression suggesting that a dead brain is not entirely dead [121]. While neuronal genes involved in brain activity are rapidly degraded, other cells and “zombie genes” come to life, making the interpretation of postmortem human brain genomic studies challenging. In fact, we found that neuronal genes are rapidly lost during the postmortem interval, yet genes involved in brain repair from glial cells including astrocytes and microglia actually increase substantially. Histological inspection at these same time points confirmed these cellular changes, as predicted, from clustering the transcriptional changes. Of note, we found that non-coding genes including microRNAs and lncRNAs were lost at a much faster rate than protein-coding genes [122]. This finding demonstrates a critical importance to studying lncRNAs in freshly isolated tissues rather than postmortem tissues, especially from the human brain.

Patients who suffer from epileptic seizures can obtain significant improvements by removing portions of their brains. Epileptic electrical networks in fact are often quite large and complex, often requiring very large portions of the brain to be removed surgically in both children and adults. In order to make critical decisions about which portions to remove and which to leave intact, extensive long-term electrical recordings are performed in vivo. When precisely localized to the electrical recordings, the brain tissues removed from these surgical procedures offer a rare opportunity to explore differences in human brain regions with abnormal and normal brain activity in freshly isolated human brain that is not affected by a postmortem interval [122].

We took a multi-systems approach to study these priceless tissues linking genomic, proteomic, and molecular differences to quantitative electrical activities, brain imaging studies, and clinical data through a relational database [123]. Previously, we found many lncRNAs that were differentially expressed as a function of human brain epileptic spiking activity from human brain biopsies at regions of low and high epileptic activities (4). One of these, BDNFOS (BDNF-AS1), was an antisense lncRNA overlapping coding portions of the Brain-Derived Neurotrophic Factor (BDNF) gene that has important roles in synaptic remodeling and neuronal survival. We found that downregulation of BDNFOS led to the upregulation of BDNF which suggested that increasing BDNFOS expression could be a way of reducing pathological BDNF signaling in epileptic brain regions. More recently, we further explored lncRNA expression and function discovering that many lncRNAs are coregulated in the human brain with known MAPK signaling pathway protein coding genes. Similar to BDNFOS, we found that some of these, particularly those antisense to nearby protein-coding genes, also regulate these coding genes, suggesting a large network of lncRNAs that can regulate important protein-coding genes within this important pathway that could lead to pathologic epileptic activities [124]. Consistently, we found that an MAPK inhibitor could prevent the development of epileptic spiking in an animal model [8].

## 8. Conclusions

This mini-review collated selected views and opinions on lncRNA biology, bioinformatics, genomics, and therapeutics, from the perspectives of several keynote, plenary and invited speakers, panelists, and organizers of the IEEE BIBM 3rd Annual LncRNA Workshop, which was held in Dubai, UAE, in December 2021. Given the focus on the 2021 Workshop, we acknowledge the limited scope of this mini-review, as it could not cover various equally important topics that were not discussed at the Workshop. Examples here include the accurate prediction of lncRNA targets and functions, context specificity of lncRNAs, lncRNAs in cell communication and immune response, connection between lncRNAs and bifunctional RNAs, etc. These provide promising avenues for future investigations.

## Data Availability

Not applicable.

## About the Authors

**Dr. Donald Adjeroh** is a Professor, Associate Departmental Chair, and Graduate Coordinator of Computer Science at the Lane Department of Computer Science and Electrical Engineering, West Virginia University (WVU), Morgantown, WV, USA. He is the Founding Director of WVU AI+Health Engineering Center. His research interests include machine learning, artificial intelligence (AI), search data structures, bioinformatics, and digital health. His research results have appeared in over 190 peer reviewed publications, including papers in several reputable conferences and journals, such as the IEEE Transactions series, top journals in theoretical computer science, and renowned journals in bioinformatics and biomedicine. Since 2019, he has served as the Founding Chair for the IEEE BIBM Annual Workshop on Long Non-Coding RNAs (BIBM LncRNA). He also served as the Program Co-Chair for the 2022 IEEE International Conference on Bioinformatics and Biomedicine (BIBM) held in two locations, Las Vegas, USA, and Changsha, China, on Dec. 6–9, 2022. Dr. Adjeroh’s research has been funded by various federal funding agencies in the USA, such as the NSF, NASA, DHS, DoD/ONR, and DoJ/NIJ, including a current US 4M NSF RII Track-2 award and another current US 3M NSF NRT project on Bridges in Digital Health, for both of which he serves as the PI. He received the Department of Energy (DOE) CAREER award in 2002, the best paper award at the 25th Workshop on Information Technology and Systems (WITS 2015), the WVU Statler Outstanding Researcher award in 2009 and 2012, the WVU Statler Excellence in Research award in 2018, 2021, and 2024, and the WVU Statler Researcher of the Year award in 2021 and 2024. Dr. Adjeroh is an Associate Editor of IEEE/ACM Transactions on Computational Biology and Bioinformatics (TCBB).

**Dr. Xiaobo Zhou** is a Fellow of the American Institute for Medical and Biological Engineering (AIMBE), a distinguished and tenured professor, and holds the Dr. and Mrs. Carl V. Vartian Professorship at UTHealth^®^ Houston, where he is the Director of the Center for Computational Systems Medicine. Dr. Zhou is a world-class scholar and expert in applying translational omics, bioimaging, medical imaging, and EMR data to precision medicine. He has published over 350 international journal articles. Per Google Scholar, as of Jan 2024, Dr. Zhou’s research has been cited over 19,000 times, and his h-index is 68. Since 2005, he has been fully and continuously funded by the NIH, NSF, and other major funding entities. He is a well-established, well-funded investigator and center director with internationally recognized expertise in basic, translational, and clinical research. Among his noteworthy accomplishments, Dr. Zhou has pioneered high-content cellular imaging informatics, bioinformatics, systems biology, systems modeling-guided cancer and regenerative medicine, and imaging-aided surgical design/optimization.

**Dr. Alexandre Paschoal** is a Group Leader in Artificial Intelligence and Informatics at the Rosalind Franklin Institute, United Kingdom, and concurrently serves as an Associate Professor at the Federal University of Technology—Parana (UTFPR), Brazil. Having earned his Ph.D. in Bioinformatics from the University of São Paulo in 2012 in Brazil, Dr. Paschoal exemplifies a commitment to interdisciplinary research. Dr. Paschoal is broadly trained in computer science, with his primary research focus lying in build cutting edge AI techniques to translate biological data into actionable knowledge utilizing principles from the field of bioinformatics. His vision involves contributions to link the fields involving data sciences and life/biological sciences to extract insights to guide decision-making. His primary biological focus lies in non-coding RNAs and transposable elements.

**Dr. Nadya Dimitrova** is an Assistant Professor in the Departments of Molecular, Cellular and Developmental Biology and Genetics at Yale University and a member of the Yale Center for RNA Science and Medicine and the Yale Cancer Center. She has a long-standing interest in understanding the functions and mechanisms of action of long non-coding RNAs (lncRNAs) in the context of cancer. Her work is focused on developing genetic models to study the roles of lncRNAs in normal and disease states and gaining deeper insights into mechanisms of lncRNAs. Nadya Dimitrova is the recipient of the HHMI Predoctoral Fellowship, the Damon Runyon Postdoctoral Fellowship Award, the Lung Cancer Research Foundation Scientific Merit Award, the Pew-Stewart Award for Cancer Research, the V Scholar Award, and the NCI Merit Award.

**Dr. Ekaterina G. Derevyanchuk** is an Associate Professor at the Genetics Department at the Academy of Biology and Biotechnology named after D.I. Ivanovsky, Southern Federal University (Rostov-on-Don, Russian Federation). She is a member of the Vavilov Society of Geneticists and Breeders. Her work focuses on the study of the role of non-coding RNAs in various diseases and the development of diagnostic test systems based on the data obtained. Particularly, her studies are related to the search of localization of ncRNA binding sites around and inside genes associated with various diseases. Dr. Derevyanchuk is a winner of the All-Russian contest, held by the Foundation for Assistance to Small Innovative Enterprises (FASIE), a German Academic Exchange Service (DAAD) scholarship holder, and a winner of the young scientists contest held within the framework of the International Congress “Early Pregnancy” (RUDN, Moscow, Russia). She is also co-owner of a small gold medal for the development of bioinformatics technology for searching for the relationship between organization scenarios in animal and human genomes of non-coding DNA and protein-coding DNA (RosBioTech, Moscow, Russia). She is the author of the best poster presentation at the Interregional Scientific and Practical Conference “Cellular Technologies for Practical Healthcare” (Yekaterinburg, Russia) and winner of the competition for the best poster report in the framework of the International Conference of Young Scientists “New directions in Life Sciences” (Yerevan, Armenia).

**Dr. Tatiana Shkurat** is Professor and Head of the Genetics Department at the Academy of Biology and Biotechnology named after D.I. Ivanovsky, Southern Federal University (Rostov-on-Don, Russian Federation). She is PI and group leader at her home institution. She has extensive experience in supervising research teams and training doctoral candidates and has led more than 30 grant-funded research projects in Russia as well as internationally sponsored projects. She has developed extensive intellectual property (IP), having been granted patents on methods of gene diagnostics of monogenic and multifactorial diseases, certificates of the computer program for automatic search of motifs, and visual analysis of images of nucleotide sequences. Recently, Prof. Shkurat has focused on the interface of non-coding DNA/RNA, evolutionary genomics, and personalized medicine. Her preliminary results represent a significant advancement toward the future deployment of approaches for the validation and utilization of novel therapeutic targets in patients with different diseases, based on lncRNA and genome editing.

**Dr. Jeffrey Loeb** is a practicing neurologist, epileptologist, and neuroscientist who currently holds the John S. Garvin Chair, Professor and Head of the Department of Neurology and Rehabilitation. He received his A.B. in Chemistry, M.D., and PhD. from the University of Chicago. After completing a residency in Neurology at the Massachusetts General Hospital in Boston, he joined the faculty of Harvard Medical School where he also had fellowship training in epilepsy at Harvard’s Beth Israel Deaconess Hospital. Dr. Loeb conducted postdoctoral work in the Department of Neurobiology at Harvard Medical School with Dr. Gerald Fischbach, where he became interested in how understanding early neural development can teach us what goes wrong in human disease and suggest new treatments. This and other work focusing on translating basic discoveries into new treatments has resulted in high-profile publications, federal and foundation grant support, and a number of patents, including one for a targeted drug that blocks neuregulin signaling and shows promise in degenerative disease by blocking neural inflammation, such as in ALS and Alzheimer’s. His work in treating epilepsy patients led to a one-of-a-kind, large-scale, systems biology project of human brain tissues that links the genes, molecular, and cellular signals that underlie the abnormal electrical activities that characterize this common neurological disorder. Dr. Loeb is director of the University of Illinois NeuroRepository, co-director of biomedical informatics in UIC’s Center for Clinical and Translational Science, and Chief Clinical Strategist for the Sturge-Weber Foundation. To build on his scientific efforts and translate discoveries back to patients through public–private partnerships, he recently formed “I-BRAIN” (Illinois Brain Analytics) in close collaboration and with support from the Discovery Partners Institute.

**Dr. Ivan Martinez** is an Associate Professor at the West Virginia University (WVU) Cancer Institute and the Department of Microbiology, Immunology and Cellular Biology at WVU School of Medicine. Originally from Mexico City, he graduated with honors from The National Autonomous University of Mexico (UNAM). He completed his Ph.D. in Molecular Genetics and Biochemistry at the University of Pittsburgh and continued his training as a postdoctoral fellow in the Department of Genetics at Yale University School of Medicine for over 5 years. After his training, he was recruited at WVU in 2013. The goal of Dr. Martinez’s research is to understand the importance of non-coding RNAs (ncRNAs), such as microRNAs (miRNAs), long non-coding RNAs (lncRNAs), and circular non-coding RNAs (circRNAs), in the process of carcinogenesis and viral infections. Dr. Martinez has been invited to present his research at prestigious institutions such as Harvard University, the University of Massachusetts Chan Medical School, the National Cancer Institute (NCI) at the National Institutes of Health (NIH), Gottingen University-Max Planck Institutes in Germany, and the National Institute of Genomic Medicine (Instituto Nacional de Medicine Genomics) in Mexico City. Since 2019, he has served as the Co-Chair for the International Conference on Bioinformatics and Biomedicine (IEEE BIBM) Annual Workshop on Long Non-Coding RNAs (BIBM LncRNA). He was also the Co-Chair in the organization of the 2022 Keystone Symposium “Small Regulatory RNAs: From Bench to Bedside” in Santa Fe, New Mexico. Dr. Martinez was also the co-leader of a team of researchers involved in the development of the COVID-19 PCR test at WVU (from in-house reagents to a fully automated testing robot). The test developed by Dr. Martinez’s team was the main test used not only at WVU but in the entire State of West Virginia during the COVID-19 pandemic. Furthermore, Dr. Martinez has been a member of the World Health Organization (WHO) COVID-19 Animal Model Group since 2021.

**Dr. Leonard Lipovich** is an internationally recognized pioneer in the long non-coding RNA (lncRNA) biology of human disease. He led a major international project with the Japan-based FANTOM Consortium (which built the first comprehensive catalog of mouse protein-coding and lncRNA genes), where he discovered that lncRNAs are often not conserved in evolution between primates and rodents and are more numerous than protein-coding genes. He built the first published catalog of human lncRNA genes supported by full-length experimental data. Leading a platform effort within the ENCODE (Encyclopedia of DNA Elements) Consortium—a direct successor to the Human Genome Project—Prof. Lipovich was the first to empirically reveal the unexpected ribosomal translation of short open reading frames from lncRNAs in human cells using mass spectrometry. His work has been fundamental to establishing that primate-specific lncRNAs in humans directly contribute to the etiology of cancer, diabetes, and epilepsy. His current focus is on identifying, and validating in the laboratory, lncRNAs from genome-wide association studies and personalized genome sequencing, as well as from in vivo transcriptome analysis and high-throughput screens, as novel and druggable causes of those diseases.

Leonard Lipovich earned his B.A. (*cum laude*) in Genetics and Development from Cornell University (1998) and his Ph.D. in Molecular Biotechnology from the Department of Genome Sciences at the University of Washington, Seattle (2003). He completed postdoctoral training at the Genome Institute of Singapore, where he characterized the first mammalian lncRNA functional in stem cell pluripotency and discovered the prevalence of primate-specific lncRNAs in sense–antisense gene pairs. Dr. Lipovich joined Wayne State University in Detroit, Michigan, USA in 2007, as an assistant professor, attaining an associate professorship and tenure in 2013, then a full professorship in 2019. In 2024, he transitioned to Wenzhou-Kean University in Wenzhou, China. He is active in lncRNA-based drug development efforts, through commercialization projects with startup RNA-biotech companies in China and elsewhere. Dr. Lipovich received the U.S. National Institutes of Health (NIH) Director’s New Innovator Award, for the 2014–2019 project cycle. In 2015, he chaired both the Keystone Symposium and the Royal Society international scientific meeting on lncRNA. To date, Dr. Lipovich (h-index: 45) has published 87 peer-reviewed papers (including 36 as a first, last, or principal author), delivered over 50 invited presentations, and chaired numerous sessions at key conferences. Dr. Lipovich is a Founding Co-Chair (2019–present) of the Annual LncRNA Workshop at the IEEE-BIBM. His goal is to improve human health through personalized, lncRNA-targeted, post-genomic therapeutics.
